# Introducing Triplex Forming Oligonucleotide into Loop-Mediated Isothermal Amplification for Developing a Lateral Flow Biosensor for Streptococci Detection

**DOI:** 10.3390/bios14050257

**Published:** 2024-05-17

**Authors:** Wei Chang, Po-Hao Chou, Cai-Tong Wu, Jheng-Da Song, Kun-Nan Tsai, Chiuan-Chian Chiou

**Affiliations:** 1 Master and PhD Program in Biotechnology Industry, College of Medicine, Chang Gung University, Taoyuan 33302, Taiwan; deamit114@gmail.com (W.C.); wendy0515w@gmail.com (C.-T.W.); chvkimo@gmail.com (J.-D.S.); 2 Delta Research Center, Delta Electronics Inc., Taipei 114501, Taiwan; pohao.ph.chou@deltaww.com (P.-H.C.); brian.kn.tsai@deltaww.com (K.-N.T.); 3 College of Biological Science and Technology, National Yang Ming Chiao Tung University, Hsinchu City 300093, Taiwan; 4 Department of Medical Biotechnology and Laboratory Science, College of Medicine, Chang Gung University, Taoyuan 33302, Taiwan; 5 Department of Laboratory Medicine, Chang Gung Memorial Hospital, Taoyuan 33302, Taiwan

**Keywords:** loop-mediated isothermal amplification (LAMP), triplex-forming oligonucleotide (TFO), lateral flow assay (LFA), *Streptococcus agalactiae*, Group B *Streptococcus* (GBS)

## Abstract

Loop-mediated isothermal amplification (LAMP) technology is extensively utilized for the detection of infectious diseases owing to its rapid processing and high sensitivity. Nevertheless, conventional LAMP signaling methods frequently suffer from a lack of sequence specificity. This study integrates a triplex-forming oligonucleotide (TFO) probe into the LAMP process to enhance sequence specificity. This TFO-LAMP technique was applied for the detection of Group B *Streptococcus* (GBS). The TFO probe is designed to recognize a specific DNA sequence, termed the TFO targeting sequence (TTS), within the amplified product, facilitating detection via fluorescent instrumentation or lateral flow biosensors. A screening method was developed to identify TFO sequences with high affinity to integrate TFO into LAMP, subsequently incorporating a selected TTS into an LAMP primer. In the TFO-LAMP assay, a FAM-labeled TFO is added to target the TTS. This TFO can be captured by an anti-FAM antibody on lateral flow test strips, thus creating a nucleic acid testing biosensor. The efficacy of the TFO-LAMP assay was confirmed through experiments with specimens spiked with varying concentrations of GBS, demonstrating 85% sensitivity at 300 copies and 100% sensitivity at 30,000 copies. In conclusion, this study has successfully developed a TFO-LAMP technology that offers applicability in lateral flow biosensors and potentially other biosensor platforms.

## 1. Introduction

Loop-mediated isothermal amplification (LAMP) is a method extensively employed in the diagnosis of infectious diseases, favored for its simplicity and efficiency. Various techniques have been devised to measure LAMP products. For example, the formation of a complex between pyrophosphate and magnesium during the amplification process is detectable via turbidimetric measurements [1]. Alternatively, the incorporation of double-stranded DNA fluorescent dyes facilitates the observation of enhanced fluorescent intensity corresponding with the amplification of double-stranded DNA [2]. Moreover, the addition of complexometric indicators (e.g., calcein) or acid-base indicators (e.g., phenol red) results in visible color changes and is perceptible to the naked eye [3,4,5,6].

Despite its benefits, LAMP’s major limitation is that the signaling methods primarily rely on nucleic acid amplification, thus lacking sequence specificity. Although the amplification is highly specific due to its requirement of at least four primers, non-specific amplification may still happen. If signaling methods only reflect double-stranded DNA amount or environmental change, it may not effectively discriminate the nonspecific amplification and lead to false-positive results [7,8,9]. In contrast, PCR methods employ sequence-specific probes, such as TaqMan, which utilize the 5′ to 3′ exonuclease activity of the polymerase for cleavage and fluorescent signal production [10]. To address this, researchers have introduced sequence-specific signal strategies into LAMP, incorporating enzymes like endonucleases [11,12,13,14], high-fidelity polymerases [15], and CRISPR-Cas proteins [16,17]. These additions enable the selective cleavage of probes bound to correctly amplified target sequences within the LAMP process. However, optimizing such reactions is challenging due to the environmental conditions required for the effective function of multiple enzymes. LAMP is susceptible to environmental fluctuations, which can impede primer binding and polymerase activity. It is the reason that initial CRISPR integrations into LAMP involved cumbersome two-step reactions [18]. Furthermore, the inclusion of additional enzymes significantly increases costs and decreases reagent stability, complicating widespread use, transportation, and storage.

To address the specificity issue in LAMP, the current study introduced a triplex form-ing oligonucleotides (TFO) probe into LAMP to generate sequence-specific signals. TFOs insert into the major groove of double-stranded DNA via Hoogsteen base pairing, forming triplex structures [19]. Based on sequence composition, TFOs are classified into poly cytosine/thymine (CT)-TFOs, poly guanine/adenine (GA)-TFOs, and poly guanine/thymine (GT)-TFOs [20]. Among these, CT-TFOs show the highest affinity but necessitate acidic conditions, contrary to the neutral to alkaline pH optimal for LAMP [20]. Given its directionalities and comparative affinity, GA-TFO was selected as the probe in this study [21].

Infections caused by *Streptococcus agalactiae* (Group B *Streptococcus*, GBS) often result in severe clinical conditions, particularly in newborns and the elderly, leading to meningitis, pneumonia, and neonatal mortality [22,23,24,25]. Thus, rapid and accurate diagnostic methods for GBS are required. Current methodologies, relying heavily on bacterial culture and biochemical identification, fall short of providing timely results. The application of LAMP for GBS detection meets the need for point-of-care diagnostic tools, thereby addressing significant public health challenges [26,27,28,29].

In the current study, we developed a novel and efficient method for GBS detection by integrating TFO probes into the LAMP framework. The LAMP reaction generates numerous repetitive sequences and TFO targeting sequences (TTSs) to which the TFO probe specifically binds. We employed fluorescently labeled probes with a quencher, observing fluorescence alterations upon probe binding and subsequent dissociation during heating. Additionally, we adapted this system for use with lateral flow strip tests, creating a compact biosensor that is both user-friendly and allows for straightforward visual interpretation of results.

## 2. Materials and Methods

### 2.1. Materials and Apparatus

The Lim broth was purchased from Becton Dickinson (Franklin Lakes, NJ, USA). The Presto™ Mini gDNA Bacteria Kit was purchased from Geneaid Biotech Ltd. (New Taipei City, Taiwan). The QIAamp DNA Micro Kit was purchased from QIAGEN (Hilden, Germany). Oligonucleotides were synthesized by Integrated DNA Technologies Biotech Co., Ltd. (Coralville, IA, USA). The oligonucleotide sequences utilized in this study are detailed in Table 1. The *Bst* 2.0 WarmStart and buffer were purchased from New England Biolabs (Ipswich, MA, USA). The nucleic acid test lateral flow strip was acquired from Panion & BF Biotech Inc. (New Taipei City, Taiwan). The other components were purchased from Sigma-Aldrich (St. Louis, MO, USA). All reagents were utilized as received and without additional purification. Melting curve analysis and LAMP were carried out on the CFX96 PCR Thermal Cycler from Bio-Rad (Hercules, CA, USA). Genomic DNA quantification was performed using EzDrop-1000 from Blue-Ray Biotech (New Taipei City, Taiwan).

### 2.2. Bacterial Genomic DNA

*Streptococcus agalactiae* (strain ATCC 13813) was cultivated in Lim broth at 37 °C overnight. Genomic DNA purification was carried out using the Presto™ Mini gDNA Bacteria Kit. Subsequently, the DNA was quantified using EzDrop-1000, and the copy number was calculated. The template was then diluted to the final concentration with double-distilled water (ddH_2_O).

### 2.3. TFO Sequence Screening Using Melting Curve Analysis

**Figure 1:**
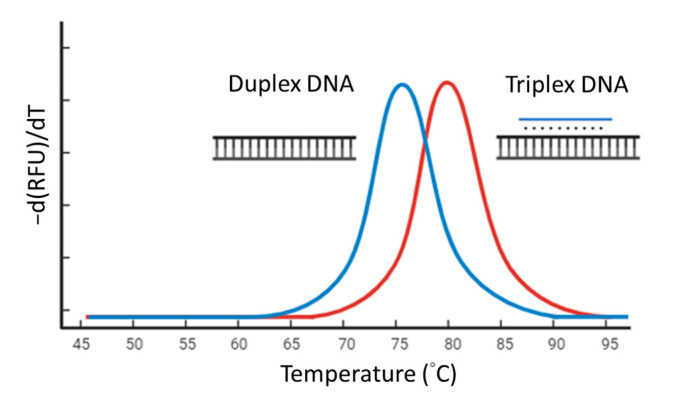
Screening high-affinity triplex-forming oligonucleotides (TFOs) with melting curve analysis. The blue peak is produced by double-stranded DNA. The red peak is produced by triplex DNA formed by the double-stranded DNA and a high-affinity TFO.

We designed 39 TFO sequences randomly. TFOs and double-stranded targets were synthesized by Integrated DNA Technologies. The melting curve analysis experiments were performed in 1 mM Tris-HCl (pH 7.5) with MgCl_2_ 4 mM and 0.5× SYBR Green I. The final concentration of the double-stranded target was 2 μM and if the TFO present was 1 μM. The mixture was gently mixed by pipetting and briefly centrifuged. Then, the mixture was heated to 95 °C for 5 min, cooled to 25 °C, and processed for melting curve analysis from 25 °C to 95 °C with continuous reading of fluorescent intensity (excitation at 483 nm and emission at 533 nm) during the temperature ramping. The negative derivative of fluorescence over temperature (−dF/dT) against the temperature change generated a curve with a peak at melting temperature (*T*m) (Figure 1). The *T*m differences between a duplex DNA and its correlated triplex DNA were determined. We constructed a model to identify the characteristics of TFO and its strength using the R programming language (R version 4.0.5) [30] with the randomForest package (version 4.6-14) [31].

### 2.4. Loop-Mediated Isothermal Amplification Assays

The SIP gene for GBS was chosen as the target template. The primer set in *sip* of GBS was designed with PrimerExplorer V5 (Eiken Chemical Co., Ltd., Tokyo, Japan), as shown in Table 1. For traditional LAMP, the reaction mixture comprised 20 mM Tris-HCl (pH 8.8), 35 mM KCl, 7 mM MgSO_4_, 10 mM (NH_4_)_2_SO_4_, 0.1% Tween 20, 0.8 M betaine, 3.2 U *Bst* 2.0 WarmStart DNA Polymerase, 1.5 mM dNTPs, 2 μL template, 1.6 μM of each inner primers (FIP and BIP), 0.8 μM of each Loop primers (LF and LB), and 0.2 μM of each outer primers (F3 and B3). To monitor the reaction, 0.25× double-stranded binding dye SYBR Green I was added. The reaction was performed at 65 °C for 45 min, and fluorescent intensity was monitored in real-time on a Bio-Rad CFX96 Thermal Cycler at 60 s intervals. The time to positive (TP) value indicated the rate of amplification. For fluorescent TFO-LAMP and melting curve analysis, one of the inner primers, BIP, was replaced with BIP_TTS which had an inserted TTS sequence, and a fluorescent TFO probe (TFO_HQ) was included at a concentration of 0.2 μM. The mixture was incubated at 65 °C for 45 min for LAMP, heated to 95 °C for 5 min, and then processed melting curve analysis from 25 °C to 95 °C. For TFO-LAMP and lateral flow assay, the loop primer LF was replaced with LF_Biotin inner primer BIP was replaced with BIP_TTS, and a FAM-labeled TFO probe (TFO_F) was added. The mixture was incubated at 65 °C for 45 min for LAMP and proceeded to lateral flow analysis.

### 2.5. Lateral Flow Assays

Following the LAMP procedure, 1 μL of the LAMP product was transferred to a tube and diluted to a final volume of 100 μL with 1× Phosphate Buffered Saline (PBS) supplemented with 5 mM Magnesium Sulfate (MgSO₄). This solution was then applied to a lateral flow test strip and incubated for 10 min. The results were subsequently assessed through visual inspection.

### 2.6. Specimen Processing

Twenty GBS-negative wound specimens were subjected to a tenfold dilution with double-distilled water (ddH_2_O). Subsequently, GBS genomic DNA was introduced into these diluted specimens. For the execution of LAMP, the specimens with spiked genomic DNA were initially heated to 95 °C for a duration of 10 min, followed by their incorporation into the LAMP reactions as templates.

## 3. Results

### 3.1. Selection of TFO Sequences

Among various types of triplex-forming oligonucleotides (TFOs), we selected the GA-TFO due to its compatibility with the buffer system utilized in the LAMP reaction. Before conducting TFO-LAMP, we first identified nucleotide sequences for TFO probes that exhibit high affinity. Our strategy involved the integration of machine learning to predict potential TFO candidates, followed by melting analysis to efficiently screen these candidates. The underlying principle of melting analysis is that a TFO of high affinity will enhance the stability of the triplex structure, thereby elevating the melting temperature (*T*m) of the triplex DNA in comparison to the duplex DNA in the absence of TFO. In this study, we randomly synthesized 39 GA-TFO probes along with their corresponding target duplexes to assess the difference in *T*m between the triplexes and the duplexes. The sequences of the TFOs and the observed *T*m differences constituted the training dataset. The reason for using this screening strategy is that if a TFO efficiently binds to its TTS and forms a stable triplex structure, the triplex structure will have a higher *T*m than the duplex DNA only. Therefore, those with a *T*m difference exceeding 5 °C were designated as efficient TFO probes (Figure 1). Various sequence features were analyzed to develop a predictive model. This model was constructed using the R programming language (Version 4.0.5) [30] and employed the Random Forest algorithm (Version 4.6-14) [31]. Subsequently, the model predicted several TFO probes deemed efficient for further validation. Following the melting curve analysis, we selected a particular TFO characterized by high affinity and minimal intra-molecular complementation for subsequent experiments (the TTS in Table 1).

### 3.2. Introducing TTS into LAMP Primer

**Figure 2:**
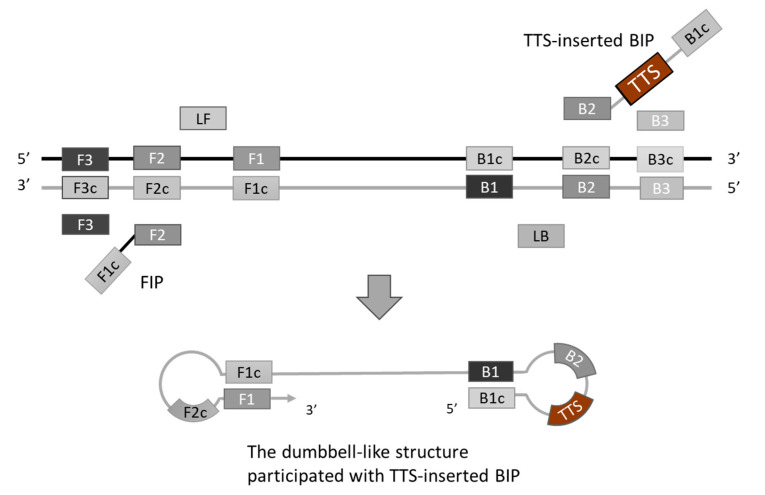
Introducing TFO targeting sequence (TTS) into loop-mediated isothermal amplification (LAMP). We inserted TTS into the middle of the inner primer BIP, between the region B1c and B2. The TTS will be part of the loop of a dumbbell-like structure.

**Figure 3:**
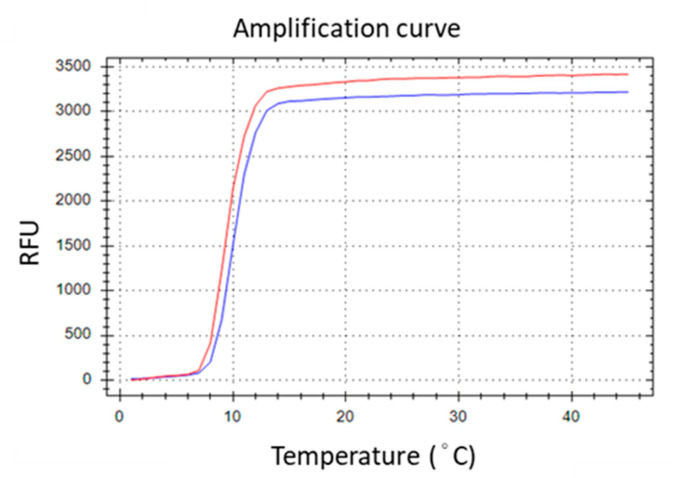
Introducing TTS into BIP primer did not remarkably affect the amplification efficiency. The red line is LAMP generated by the original primer set. The blue line is that generated by the primer set except the BIP was replaced with the TTS-inserted BIP.

To generate TFO binding sites on the LAMP amplicons, the selected TTS was integrated into the LAMP primers. Various positions across different primers were experimentally assessed, with the optimal insertion point for the TTS at the central region of the inner primer, between the B1c and B2 segments. This insertion strategy resulted in the formation of a larger dumbbell-like structure of the loop (Figure 2). Subsequently, the amplification efficiency was evaluated by comparing the performance of the original inner primer against that modified to include the TTS. The comparative analysis revealed no significant difference in LAMP efficiency, suggesting that the incorporation of TTS within the inner primer exerts minimal impact on the amplification process (Figure 3).

### 3.3. Producing Fluorescent Signal Using a TFO Probe in LAMP

**Figure 4:**
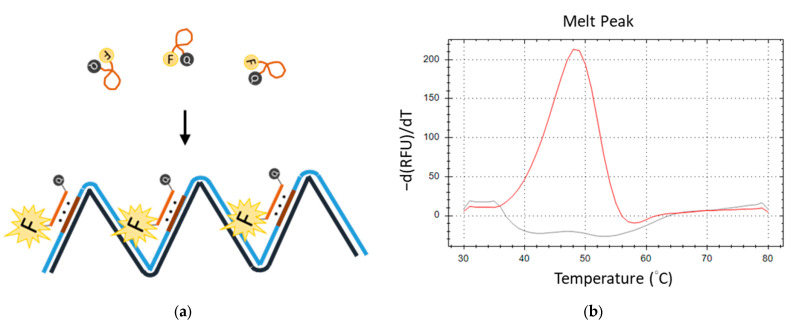
The fluorescent melting peak generated by a TFO probe. (**a**) TFO probe, which is labeled with a fluorescent reporter (F) and quencher (Q), produces a fluorescent signal when the correct LAMP product is generated. (**b**) The melting peak produced by the fluorescent TFO probe and LAMP products is shown as the red line. That by the fluorescent TFO in no template control is shown as the gray line.

Subsequently, we engineered a TFO probe featuring self-quenching fluorescence labels, which comprised a fluorescent reporter at its 5′ end and a dark quencher at the 3′ end. The LAMP reaction that comprised a primer with TTS and a TFO probe was named TFO-LAMP. The LAMP reaction can produce many TTSs in its product. The TFO probe can attach to the TTSs of the LAMP products. During amplification, the TFO probes and the TTSs formed triplex structures. This conformational change extends the average distance between the fluorescent reporter and the quencher beyond that in the free probe state, resulting in an enhanced fluorescence signal (Figure 4a). Melting curve analysis subsequently enables the detection of a distinct melting peak corresponding to the triplex formation, with ’he peak’s temperature (equal to its *T*m) serving as an indicator of the TFO’s binding affinity (Figure 4b). Utilizing feedback from the melting curve analysis, we refined the reaction conditions by optimizing the concentrations of potassium chloride to 35 mM and magnesium chloride to 7 mM, thereby ensuring the efficacy of both the LAMP reaction and TFO binding.

### 3.4. Lateral Flow and Melting Results

We subsequently advanced the TFO-LAMP methodology by incorporating it into a lateral flow strip biosensor, facilitating straightforward visual interpretation. The TFO probe was conjugated with fluorescein amidite (FAM), and biotin was appended to one of the loop primers. Upon binding of the probe to the LAMP product, the resultant complex carried both FAM and biotin labels.

**Figure 5:**
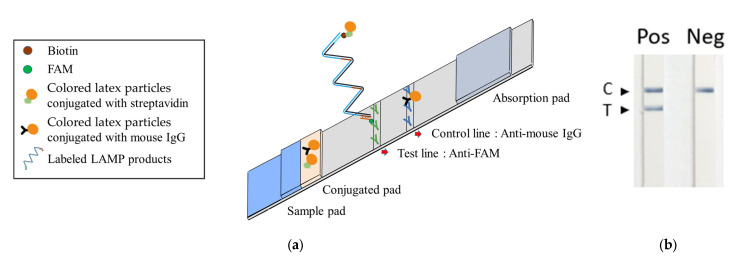
Integrated TFO-LAMP onto a lateral flow strip biosensor. (**a**) Principle of TFO-LAMP lateral flow assay. The probe-LAMP product complexes with colored streptavidin latex can be trapped on the test line and generate a visible band. The control beads can bind to the control line. (**b**) TFO-LAMP results on lateral flow strips. In the positive (Pos) group, the TFO-LAMP reaction contained 100 pg GBS genomic DNA as a template. In the negative (Neg) group, the TFO-LAMP reaction contained no template. C, control line. T, test line.

The lateral flow strip developed for the TFO-LAMP assay operates on the immunochromatographic principle. The conjugation pad is impregnated with colored latex particles, which are conjugated with streptavidin or mouse IgG. At the central region of the device, the nitrocellulose membrane features both a test and a control line. The test line is coated with anti-FAM antibodies, while the control line is coated with anti-mouse IgG antibodies (Figure 5a). The LAMP reaction with BIP_TTS primer and LF_biotin primer generated amplicons that interacted with the strip (Figure 5a). Firstly, the FAM-labeled TFO probes bound to TTSs on the amplicons, which could be captured by the anti-FAM antibody on the test line. Secondly, the biotin on the amplicon could attract streptavidin-latex particles. Therefore, the interaction between the LAMP amplicons, TFO probes, anti-FAM antibodies, and latex particles caused an accumulation of colored latex on the test line, manifesting as a visible signal. Simultaneously, colored latex particles conjugated with mouse IgG are captured on the control line by the anti-mouse IgG, functioning as an internal quality control to verify the integrity of the lateral flow strip and confirm the successful application of the sample (Figure 5a).

**Figure 6:**
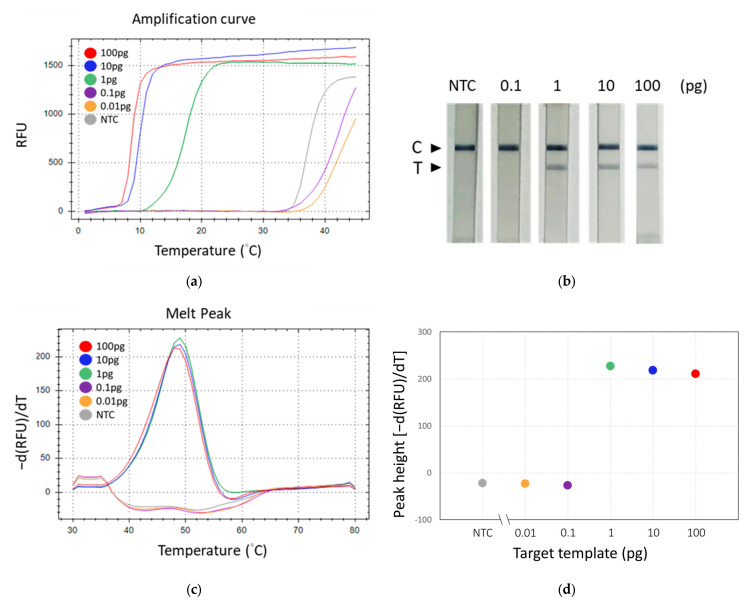
Evaluation of the detection sensitivity of TFO-LAMP. Serial dilutions of GBS genomic DNA were analyzed using three different methods. (**a**) Fluorescent amplification curves generated by the LAMP products and double-stranded binding dye, SYBR green I. (**b**) Results on lateral flow strip generated by the LAMP products and FAM-labeled TFO probe. (**c**) Melting curves generated by the LAMP product and HEX-labeled TFO probe. (**d**) Dose-response curve derived from melting peaks. C, control line. T, test line. NTC, no template control.

Observations revealed that the group supplemented with GBS genomic DNA (positive) exhibited a band on the test line, in contrast to the group devoid of added DNA (negative), which did not display such a band (Figure 5b). Nonetheless, both groups manifested bands on the control line. Subsequent experiments entailed the performance of tenfold serial dilutions of GBS DNA to assess analytical sensitivity. In these experiments, we employed three different methods to detect the TFO-LAMP products: double-stranded DNA binding dye SYBR Green I, HEX fluorophore-labeled TFO probe, and FAM-labeled TFO probe combined with lateral flow assay (Figure 6). The double-stranded DNA binding dye generated amplification curves for the LAMP products (Figure 6a), while the FAM-labeled probes and lateral flow strips yielded visible lines (Figure 6b), and the HEX-labeled probes generated melting peaks (Figure 6c). The dose-response curves derived from melting peaks are shown in Figure 6d. Since the end-point results of LAMP were all-or-none manner, the positive reaction showed a melting peak higher than 200 (Figure 6d). The convergence of results from these three methodologies indicated a limit of detection (LoD) of approximately 1 pg of GBS genomic DNA, corresponding to roughly 300 genomic copies. These outcomes demonstrate that both the amplification curve and the melting peak accurately mirror the results observed on the lateral flow strips, suggesting that the deployment of lateral flow strips could eliminate the necessity for sophisticated equipment.

### 3.5. The Detection of Spiked Specimens

The feasibility of the TFO-LAMP and lateral flow biosensor was validated with spiked specimens. Twenty wound samples, verified to be devoid of GBS infection, served as the matrix for this validation exercise. Each sample underwent a tenfold dilution and was subdivided into three aliquots. Varied quantities of GBS genomic DNA were subsequently introduced into these aliquots. These simulated specimens were subjected to a thermal treatment at 95 °C for 10 min, followed by direct incorporation into the TFO-LAMP reaction, bypassing the step of nucleic acid purification. Post-LAMP, the reaction products were diluted by a factor of 100 and applied to lateral flow strips. The analytical outcome indicated that all twenty specimens, devoid of bacterial DNA, were correctly identified as negative by the biosensor, with no incidence of false-positive results recorded. In the subset spiked with 30,000 copies of GBS genomic DNA, detection was uniformly positive, reflecting a sensitivity rate of 100%. Conversely, in the subset spiked with 300 copies, 17 samples were positively identified, yielding a sensitivity rate of 85% (Table 2). These results affirm the high sensitivity and specificity of the devised biosensor in processing simulated specimens.

## 4. Discussion

In this investigation, TFOs were employed as innovative probes to facilitate sequence-specific recognition of TTSs within LAMP amplicons. Through strategic labeling, TFO probes were designed to either produce a fluorescent signal detectable by real-time fluorometers or to bind to antibodies immobilized on a lateral flow strip, rendering them suitable for diverse clinical diagnostics.

One notable advantage of LAMP is its remarkable robustness against biological interferences, which permits the bypassing of the nucleic acid purification step, thus simplifying and expediting the diagnostic workflow. Comparing Figure 6 and Table 2, we found that the detection limits in both experiments were around 300 copies, or 1 pg genomic DNA, indicating that using the DNA spiked in wound samples did not reduce sensitivities and that the 1:10 dilution of the sample had lesser matrix effects. While the sensitivity of LAMP may be marginally lower than that of PCR, LAMP compensates with a significantly streamlined process. It eliminates the necessity for laborious and costly purification systems and does not require an elaborate thermal cycler. Such characteristics render LAMP particularly beneficial in scenarios demanding swift diagnostic results, such as in the timely screening of GBS in expectant mothers and newborns, providing them with prompt test confirmations. Also, since the results can be observed directly with the naked eye, the demand for sophisticated instruments is greatly reduced. Therefore, combining isothermal amplification with lateral flow strip testing is very suitable for use in resource-limited areas.

The increased specificity of TFO-LAMP is achieved through the following points: First, the TTS exists only on one primer, so if this primer does not participate in the generation of non-specific products, the binding of the TFO to the correct amplicon is specific. Second, in the lateral flow assay, only the amplified products involving the primers BIP_TTS and LF_biotin can form a positive reaction at the test line, and the amplified products formed by these two primers are highly likely to be the correct products. Compared to another sequence-specific detection method for LAMP products, namely mediator displacement LAMP [32,33], our TFO probe offers superior compatibility for integration into biosensors. The mediator displacement LAMP technique relies on a zipper probe mechanism, wherein the probe dissociates from the amplicon as amplification progresses [34]. In contrast, our TFO probe remains associated with the amplicon throughout the amplification process, providing a stable and continuous signal for biosensor applications. This fundamental difference in probe-amplicon interaction dynamics underscores the enhanced suitability of TFO probes for use in biosensor-based diagnostics.

Moreover, our probe’s design enables swift incorporation into diverse LAMP reactions through the simple exchange of primer sequences to embed TTS, markedly diminishing both the development costs and time. We have also established an effective method for the screening and evaluation of TFO sequences, identifying candidates demonstrating high affinity. These features facilitate designs of multiplex reactions, as multiple target-probe pairs can be easily generated by the streamline. By labeling with different tags on the TFO probes, different LAMP targets can be differentiated by colors or positions on a biosensor.

However, using TFO probes presents some potential limitations: Firstly, it is difficult to find good TTSs in the bacterial genomes. Therefore, a TTS must be introduced into the LAMP amplicons via designed primer, as did in the current study. Secondly, the G-rich TFO is prone to generate G-quadruplex structures. The G/A allocation and salt concentrations need intensive optimization to avoid the intra-molecular structure in TFO.

The demonstrated efficacy of the TFO probe in conjunction with lateral flow strips, a type of solid-phase biosensor, highlights its adaptability to a variety of biosensing platforms. Furthermore, this success intimates the feasibility of its seamless integration into microfluidic chip technologies, potentially broadening the scope of its application in rapid diagnostic and analytical tools.

## 5. Patents

We have filed a patent “Detection kit and detection method for detecting target sequence”.

## Figures and Tables

**Table 1:** Oligonucleotide sequences.

Oligonucleotide	Sequence 5′-3′
FIP	AACACCAGCCATCATAACTCCTAGTCATTCCTGCTCTACCACTT
BIP	TGGGAGGAAGAAAAGGGAGTTTATTCGAACGTAAACCTGAGAA
F3	GATGGCTATGATGGCTACT
B3	GCTGTTGGTCCTGTCAAT
LF	AGGGACTGGAATGAAACC
LB	TTACTCTTCTTTCTTGTCGG
LF_Biotin	Biotin-AGGGACTGGAATGAAACC
BIP_TTS	TGGGAGGAAGAAAAGGGAGTTTATGGAAGAAGAAGAGAAGAAGGAGAGGTCGAACGTAAACCTGAGAA
TFO_HQ	HEX-GGAGAGGAAGAAGAGAAGAAGAAGG-BHQ1
TFO_F	GGAGAGGAAGAAGAGAAGAAGAAGG-FAM

Notes: The underlined region represents the TFO targeting sequence inserted into the inner primer.

**Table 2:** The lateral flow results in simulated specimens.

	Positive/Sample No.	Sensitivity
Spike in 30,000 copies	20/20	100%
Spike in 300 copies	17/20	85%
negative	0/20	-

## Data Availability

No new data were created.
